# Prevalence of CD30 immunostaining in neoplastic mast cells: A retrospective immunohistochemical study

**DOI:** 10.1097/MD.0000000000010642

**Published:** 2018-05-25

**Authors:** Geisilene Russano de Paiva Silva, Emilie Tournier, Luis Otávio Sarian, Cristina Bulai-Livideanu, Georges Delsol, Laurence Lamant, José Vassallo, Pierre Brousset, Camille Laurent

**Affiliations:** a UMR U.1037, Centre de recherche sur cancer de Toulouse, Université Paul-Sabatier; b Department of Pathology, Institut Universitaire du Cancer de Toulouse-Oncopole; c Department of Dermatology, Paul Sabatier University, Mastocytosis National Reference Center (CEREMAST), Toulouse University and CHU, Toulouse, France; d Laboratory of Molecular and Investigative Pathology—LAPE, Faculty of Medical Sciences, State University of Campinas Medical School, Campinas, Brazil; e Laboratoire d’excellence Labex TOUCAN, Toulouse, France.

**Keywords:** CD30, cutaneous mastocytosis, immunohistochemistry, mastocytosis

## Abstract

Mastocytosis is a rare disease characterized by clonal neoplastic proliferation of mast cells (MCs). It ranges from skin lesions as cutaneous mastocytosis (CM) which may spontaneously regress to highly aggressive neoplasms with multiorgan involvement corresponding to some aggressive systemic mastocytosis (ASM), mast cell leukemia (MCL), and/or mast cell sarcoma (MCS).

There is increasing evidence of CD30 expression in neoplastic MCs of the bone marrow. This expression has been described almost exclusively in aggressive forms of systemic mastocytosis (SM).

The aim of the present study is to evaluate CD30 expression both in cutaneous and systemic forms of mastocytosis. Forty-two mastocytosis cases were reviewed, including cutaneous (n = 29) and systemic (n = 13) forms to assess the prevalence of CD30 expression. Thirty-nine out of 42 (92.8%) cases were CD30 positive. In cases of CM, 28/29 (96.5%) cases were CD30 positive, 11/13 cases of SM (84.6%) were positive for CD30. MCs in normal skin biopsies and in urticaria lesions were CD30-negative. This study found that CD30 is also frequently expressed in CM as well as in systemic forms. This finding is a major departure from the prevailing concept that CD30 expression is often related to aggressive systemic forms of mastocytosis.

## 1 Introduction

Mastocytosis is a rare disease with an estimated frequency of 1:1000–8000.^[1]^ Mastocytosis is defined as a clonal accumulation/proliferation of mast cells (MCs) that infiltrate one or more organs. The etiology of the disease remains unknown and its manifestations are heterogeneous, ranging from isolated skin lesions that may spontaneously regress as cutaneous mastocytosis (CM) to highly aggressive neoplasm associated with multivisceral involvement and sometimes with short survival times found in aggressive systemic mastocytosis (ASM), mast cell leukemia (MCL), and mast cell sarcoma (MCS).^[2]^

The diagnosis of mastocytosis is based on the histopathologic demonstration of clusters of neoplastic MCs in the involved organ. After the histologic diagnosis, the different variants of mastocytosis can be recognized by applying the World Health Organisation (WHO) 2017 criteria for therapeutic and prognostic purposes.^[3]^

The major criterion for SM is the presence of multifocal dense aggregates of ≥15 MCs detected in sections of bone marrow and/or other extracutaneous organs. The minor criteria are: Atypical morphology or spindle shapes in >25% of the MCs in bone marrow sections, bone marrow aspirate, or other extracutaneous tissues; mutational analysis of Kit showing a 816 mutation codon (e.g., Asp816Val) in bone marrow, blood, or extracutaneous organs; bone marrow or other extracutaneous MCs expressing the surface markers CD2, CD25, or both; Baseline serum tryptase levels >20 ng/mL. The final diagnosis of SM will be rendered if the major criterion plus one of the minor criteria or 3 minor criteria are fulfilled.^[3]^

Immunomarkers are useful tools for the diagnosis of mastocytosis because sometimes it may be difficult to discriminate between true mastocytosis and MCs hyperplasia.^[3]^

MCs express CD117 and tryptase antigens^[4]^ and may exhibit CD63 and CD69 activation-associated antigens.^[5]^ Others markers related to complement-related cells surface antigens are expressed in a high proportion of SM cases, for example, CD11b/CR3, CD11c/CR4, CD35/CR1, CD55/DAF, CD59/MIRL, and CD88/C5aR.^[5]^

CD30 is a transmembrane glycoprotein belonging to the tumor necrosis factor superfamily. CD30 is expressed in activated or proliferating B and T cells, but it is absent from or very weak in normal tissues. Expression of CD30 has been demonstrated in non-lymphoid and lymphoid neoplasms, like Hodgkin lymphoma.^[6]^ A recent work has demonstrated that CD30 is frequently expressed in the aggressive form of mastocytosis raising the hypothesis of a specific association.^[7–10]^ However, the study by Morgado et al^[11]^ using flow cytometry analysis showed that CD30 expression in bone marrow MCs was detected in both aggressive and indolent disease.

Due to the clinical impact of the differential diagnosis between indolent (as CM) and aggressive forms of mastocytosis, this retrospective study proposes to evaluate the presence of CD30 immunomarker in a series of MCs lesions.

## 2 Materials and methods

### 2.1 Inclusion criteria

We retrieved, from medical files of the Pathology Department of CHU Purpan (Toulouse, France), clinical, and pathological data from 42 mastocytosis cases treated from 2000 to 2006. Tissue samples were collected and processed following standard ethical procedures (Helsinki Declaration).

The histopathological slides were reviewed by two senior pathologists that are specialized in skin diseases. CD30 and CD117 immunohistochemical analysis was additionally performed in all cases. The histopathological analysis and the CD30 immunostaining were also done in a control group comprising of 5 normal skin samples from various parts of the body (retrieved from plastic surgery procedures) and 16 cases of urticaria.

### 2.2 Histopathological analysis

The skin biopsies from mastocytosis were fixed in formalin solution (3 cases) and the remaining 39 cases were fixed in Bouin's solution. After paraffin embedding, 4 μm thick tissue sections were stained with hematoxylin and eosin, Giemsa, and toluidine blue. The urticaria skin control lesions were fixed in Bouin's solution (9 cases) and 7 cases were fixed with formalin solution. The 5 cases of normal skin control were fixed with Bouin's solution (3 cases), and formalin solution in 2 cases.

### 2.3 Immunohistochemistry

For immunohistochemistry, 3-μm-thick sections were tested using a Ventana BenchMark XT immunostainer (Ventana, Tucson, AZ). Immunohistochemistry using avidin–biotin complex was performed using the panel of the following antibodies: CD30 (BerH2, 1:50; Dako), CD117 (c-Kit) (A4502, 1:100; Dako) and in 25 patients CD2 immunostaining (AB75, 1:50; Novocastra) was performed. In one patient with atypical presentation, a larger panel of immunomarkers was performed (not shown).

The intensity of CD30 and CD117 immunostaining was graded as: negative (0), weak (+), intermediate (++) and strong (+++). The percentage of CD30 positive cells examined was defined as negative, low (<10%), intermediate (10%–50%), and high (>50%).

### 2.4 Molecular analysis for the detection of c-Kit mutation

In one case of systemic mastocytosis, due to its atypical immunohistochemical presentation (positive CD30), a molecular analysis for the detection of c-Kit mutation was carried out.

Total RNA was extracted from frozen skin biopsies using the RNeasy mini kit (Qiagen, Courtaboeuf, France). Complementary DNA was synthesized by using random hexamers as a primer from 200ng of total RNA. ProStar First-Strand RT-PCR kit (Stratagene, La Jolla, CA) was used, consuming a total volume of 25 μL as recommended by the manufacturer. Then, 2.5 μL of cDNA was introduced in each PCR reaction. The c-Kit gene was amplified by polymerase chain reaction (PCR) using HotStarTaq DNA polymerase (Qiagen, Courtaboeuf, France). A total of 40 cycles were performed using the Gene Amp PCR System 2700 or 9700 (Applied Biosystems, Courtaboeuf, France) at 94°C for 30 seconds, 57°C for 30 seconds, and 72°C for 45 seconds. The sequences of c*-*Kit coding were amplified from complementary DNA with the PCR by using primer pairs indicated in Table 1. Direct amplimer sequencing was carried out after the purification of the PCR products with the Minelute PCR purification kit (Qiagen). They were directly sequenced with BigD dye terminator V 1.1 (Applied Biosystems) and an ABI PRISM 3100 sequencer (Applied Biosystems) and analyzed with the Seqscape software (Applied Biosystems). D816V mutation (exon 17) was also tested by restriction digest analysis with BsmA1 and Ple1 restriction enzymes that detect wild types or mutated forms respectively. Fluorescent primers (U2F and L1F, see Tables 1 and 2) were used for PCR reactions and size of restriction digest fragments were directly determined on a 16 capillary sequencer (ABI Prism 3100 sequencer) with the GeneMapper software (Applied Biosystems). Primer positions are indicated by the c-Kit sequence published through the NCBI accession number X06182.

**Table 1:**
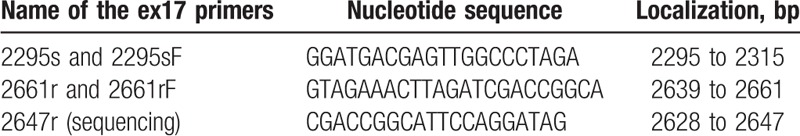
Primer positions are indicated by the c-Kit sequence published through the NCBI accession number X06182.

**Table 2:**
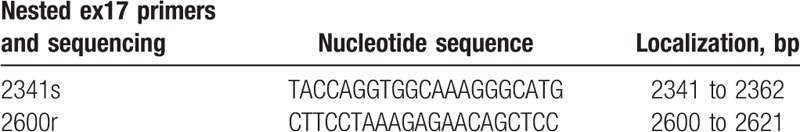
Primer positions are indicated by the c-Kit sequence published through the NCBI accession number X06182.

## 3 Results

### 3.1 Clinical patients

Data from 42 cases of mastocytosis were retrieved from the files. Twenty-nine cases corresponded to CM and 13 cases to SM according to the WHO 2017 criteria.^[3]^ Concerning CM, the patients’ ages varied between 2 and 13 years old in 11 cases and in 18 cases there was a wide range between 25 and 78 years old. In cases of SM, the ages varied between 31 and 85 years old.

We have follow-up data for 28 of the 29 CM cases. Up until now, none of these patients developed SM.

### 3.2 CD117 immunostaining in neoplastic and reactive MCs

In CM and SM, the neoplastic MCs were strongly positive for CD117 in 100% of MCs. There was no difference in intensity of staining between CM and SM. Neoplastic cells were distributed diffusely in the dermis. In normal and urticaria skin biopsies, all reactive MCs were CD117 positive but with a weak staining. These cells were rare and scattered compared to neoplastic MCs counterpart and were almost always located surrounding blood vessels.

### 3.3 CD30 immunostaining in neoplastic and reactive MCs

Out of the 42 cases of mastocytosis, 39 cases (92.8%) were positive for CD30. In two cases CD30 was not readable (SM). In one case CD30 was negative (CM).

**Figure 1:**
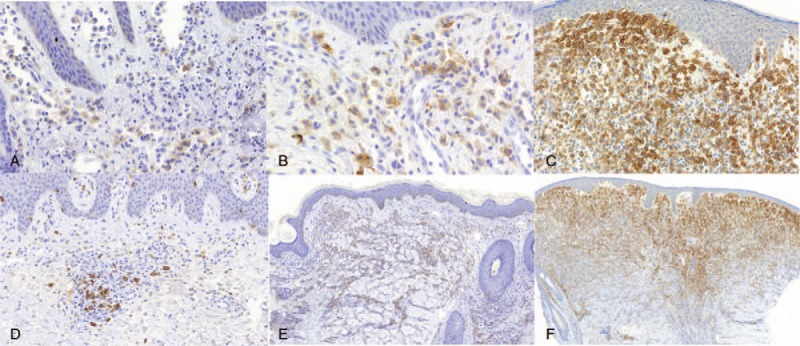
Progressive CD30 intensity and extension in infiltrates of neoplastic MCs in cases of CM. (A) Weak CD30 immunostaining, magnification ×400. (B) Intermediate CD30 immunostaining, magnification ×400. (C) Strong CD30 immunostaining, magnification ×200. (D) CD30 staining in <10% of MCs, magnification ×200. (E) CD30 staining between 10% and 50% of MCs, magnification ×100. (F) CD30 staining of more than 50% of MCs, magnification ×50. MCs = mast cells, CM = cutaneous mastocytosis.

Except for one case, all CM cases (n = 28/29) were CD30 positive (96.5%). In 16 CM, CD30 staining was positive in 10% to 50% MCs and in 11 cases CD30 was positive in more than 50% of MCs. In only one case of CM, CD30 was positive in <10% of MCs. Regarding the intensity of CD30 immunostaining in CM forms 16 cases were graded as weak, 8 cases were intermediate, and 4 cases were strongly positive (Fig. 1).

Of the 28 CM cases positive for CD30, 22 presented clinically as urticaria pigmentosa (UP)/maculopapular cutaneous mastocytosis (MPCM), 2 cases as diffuse cutaneous mastocytosis and 2 cases as solitary mastocytoma of skin. In the two remaining cases, there was no clinical information about the CM subtype. CD30 staining was positive: in more than 50% of MCs in 7 cases of UP/MPCM, in one case of diffuse cutaneous mastocytosis and for 2 cases of solitary mastocytoma of skin; CD30 staining between 10% and 50% of MCs occurred in 14 cases of UP/MPCM and in one case of diffuse cutaneous mastocytosis; c) CD30 staining of < 10% of MCs was found in only one case of UP/MPCM. The CD30 immunostaining intensity was graded as: weak in 14 cases of UP/MPCM and in one case of solitary mastocytoma of skin, moderate in 6 cases of UP/MPCM, in one case of diffuse cutaneous mastocytosis and mastocytoma of skin each, strong in 2 cases of UP/MPCM and in one case of diffuse cutaneous mastocytosis.

**Figure 2:**
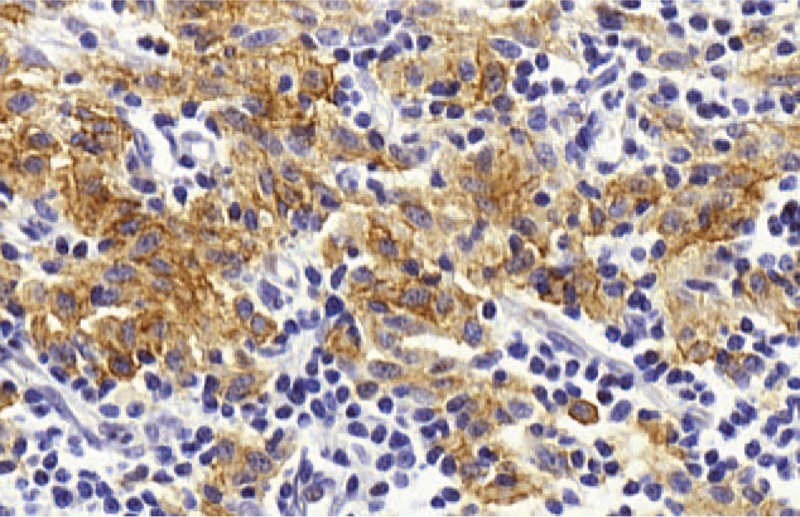
Strong CD30 immunostaining in MCs from a case of SM, magnification ×600. MCs = mast cells, SM = systemic mastocytosis.

All tested cases of SM (n = 11/13) were positive for CD30 with a CD30 staining ranged from 10% to 50% of MCs in 7 cases and with a CD30 staining in more than 50% of MCs in 3 cases. The intensity of CD30 immunostaining in the SM cases was weak in 9 cases, intermediate in 1 case and strong in the latter case (Fig. 2).

**Figure 3:**
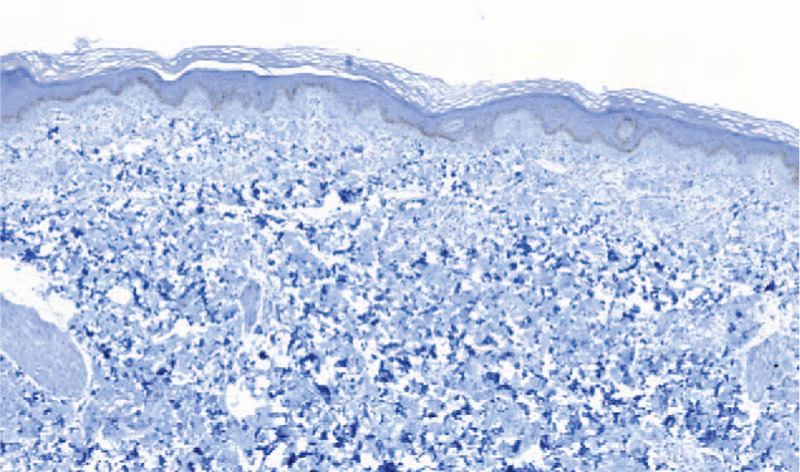
An urticaria lesion negative for CD30 immunostaining, magnification ×40.

By contrast, the reactive MCs of normal skin biopsies (n = 5) did not express CD30. Likewise, CD30 was also negative in reactive MCs of urticaria skin lesions (n = 16) (Fig. 3).

CD30 positivity was found in both fixative groups (Bouin and Formalin).

Demographic data, mastocytosis subtypes and immunohistochemical results of all 42 patients are shown in Table 3.

**Table 3:**
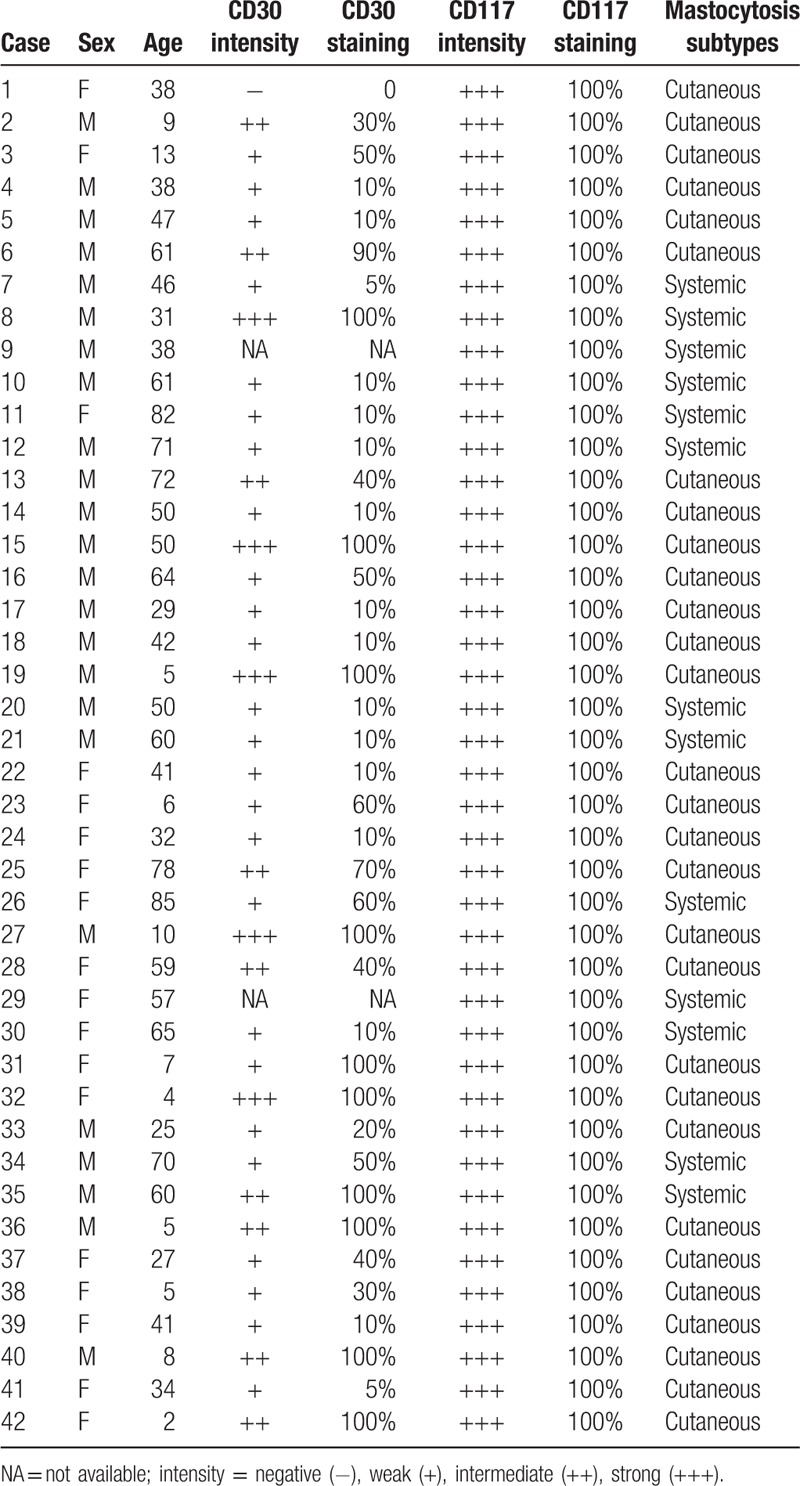
Cases demography, immunohistochemical results and their distribution related to the subtype of mastocytosis subtype.

### 3.4 Molecular analysis for the detection of c-Kit mutation

Due to a very atypical morphological presentation in one case of SM, the presence of the D816V mutation was searched and established. That unexpected case led to the retrospective research of CD30 immunostaining in all the other cases.

## 4 Discussion

Mastocytosis is a rare disease^[1]^ with a highly heterogeneous behavior that varies in which skin lesions could spontaneously regress or become an aggressive neoplasms with pejorative prognosis.^[2,3,12]^ Due to this extremely variable behavior of mastocytosis, an important question is to identify patients with favorable outcome from those with pejorative prognosis despite of the standard therapies. The immunohistochemical studies might play a role in the diagnosis process of atypical cases and could help to identify mastocytosis patients with pejorative outcome.

Different suitable immunohistochemical markers in histopathology routine have been proposed for the identification of neoplastic MCs. Examples include tryptase and other MCs-related antigens such as chymase, CD117, CD68, CD2, and CD25. In addition to these markers, CD30 could also be useful especially in atypical mastocytosis cases as illustrated in our series. The CD30 expression in occasional MCs, as previously reported,^[13,14]^ led us to do a retrospective search for CD30 expression in mastocytosis.

CD30 is considered a member of the tumor necrosis factor receptor superfamily and found in neoplastic cells as embryonal carcinoma, Hodgkin lymphoma, anaplastic large cell lymphoma, and in some cases of extramedullary myeloid sarcoma.^[7]^

Sotlar and Valent^[7,15]^ were able to demonstrate CD30 immunostaining in patients having ASM and MCL and they postulated that CD30 could be a marker of worse prognosis. They also demonstrated low or even absent CD30 expression in patients with indolent mastocytosis.

The detection of CD30 in neoplastic MCs in indolent forms of mastocytosis have been rarely described.^[11]^ Although there is no reliable biomarker that correlates with the prognosis, a possible relationship between CD30 expression and a worse outcome has been described almost exclusively in aggressive forms of SM.^[7]^ To investigate the prognosis value of CD30 in mastocytosis, we tested CD30 immunostaining in a retrospective series of CM and SM patients.

Our results showed that CD30 expression was present in the majority of mastocytosis cases independently of their systemic and cutaneous presentation. However, CM cases showed a higher percentage of CD30 positive cells than SM. In addition, MCs in CM showed a higher intensity of CD30 staining with a stronger expression of this marker in the 14.2% of CM cases as compared to those found in SM. This finding deviates from those reported previously, in which prevailed negative or infrequent CD30 immunostaining in cases of CM.^[7,16,17]^

The significance of tumoral CD30 expression is yet a matter of debate. CD30 expression can be associated with a malignant phenotype, or reflect the recruitment of an inflammatory milieu that enhances tumor growth and survival.^[18]^ One could argue that CD30 positivity in CM might be the result of MCs activation or allergy and not be related to CM oncogenesis. In order to answer to that question, we tested both normal skins and urticaria lesions, which were CD30 negative nonetheless.

One could speculate that CD30 may be involved in a specific signalling pathway in neoplastic MCs and could be a potential therapeutic target in mastocytosis. Indeed, phase I and II trials^[18]^ demonstrated that the monoclonal antibody brentuximab vedotin yielded meaningful control of Hodgkin Lymphoma, peripheral T-cell Lymphoma, cutaneous T-cell Lymphoma, and even in cases of SM.^[10]^ These findings illustrate that CD30 is a potential target for the treatment of mastocytosis.

## 5 Conclusion

In this study, CD30 was found to be frequently positive in mastocytosis regardless of its clinical presentation. However, we found a higher percentage of positive MCs and stronger staining intensity in CM subtypes. These findings suggest that CD30 is a marker of neoplastic MCs and not exclusively present in aggressive forms of the disease as previously reported.

## Author contributions

**Conceptualization:** Geisilene Russano de Paiva Silva, Emilie Tournier, luis Otavio Sarian, Cristina Bulai-Livideanu, Georges Delsol, Laurence Lamant, Jose Vassallo, Pierre Brousset, Camille Laurent.

**Formal analysis:** Geisilene Russano De Paiva Silva, Emilie Tournier, Luis Otavio Sarian, Cristina Bulai-Livideanu, Georges Delsol, Laurence Lamant, Jose Vassallo, Pierre Brousset, Camille Laurent.

**Investigation:** Geisilene Russano De Paiva Silva.

**Methodology:** Geisilene Russano De Paiva Silva, Emilie Tournier, Luis Otavio Sarian, Cristina Bulai-Livideanu, Georges Delsol, Laurence Lamant, Jose Vassallo, Pierre Brousset, Camille Laurent.

**Supervision:** Luis Otavio Sarian, Cristina Bulai-Livideanu, Georges Delsol, Laurence Lamant, Jose Vassallo, Pierre Brousset, Camille Laurent.

**Validation:** Geisilene Russano De Paiva Silva, Emilie Tournier, Luis Otavio Sarian, Cristina Bulai-Livideanu, Georges Delsol, Laurence Lamant, Jose Vassallo, Pierre Brousset, Camille Laurent.

**Visualization:** Geisilene Russano De Paiva Silva, Emilie Tournier, Luis Otavio Sarian, Cristina Bulai-Livideanu, Georges Delsol, Laurence Lamant, Jose Vassallo, Pierre Brousset, Camille Laurent.

**Writing – original draft:** Geisilene Russano De Paiva Silva.

**Writing – review & editing:** Geisilene Russano De Paiva Silva, Emilie Tournier, Luis Otavio Sarian, Cristina Bulai-Livideanu, Georges Delsol, Laurence Lamant, Jose Vassallo, Pierre Brousset, Camille Laurent.
